# Hearing Loss in Baraitser–Winter Syndrome: Case Reports and Review of the Literature

**DOI:** 10.3390/jcm13051500

**Published:** 2024-03-05

**Authors:** Sara Ghiselli, Giulia Parmeggiani, Giulia Zambonini, Domenico Cuda

**Affiliations:** 1 Department of Otorhinolaryngology, AUSL Piacenza, 29121 Piacenza, Italy; g.zambonini@ausl.pc.it (G.Z.); d.cuda@ausl.pc.it (D.C.); 2 Medical Genetics Unit, AUSL Romagna, 47522 Cesena, Italy; giulia.parmeggiani@auslromagna.it; 3 Department of Medicine and Surgery, University of Parma, 43121 Parma, Italy

**Keywords:** hearing loss, Baraitser–Winter Syndrome, ACTB gene, ACTG1 gene, Autosomal dominant non-syndromic hearing loss 20/26

## Abstract

**Background**: Baraitser–Winter Syndrome (BRWS) is a rare autosomal dominant condition associated with hearing loss (HL). In the literature, two types of this condition are reported, Baraitser–Winter type 1 (BRWS1) and type 2 (BRWS2) produced by specific pathogenetic variants of two different genes, ACTB for BRWS1 and ACTG1 for BRWS2. In addition to syndromic BRWS2, some pathogenic variants in ACTG1 are associated also to another pathologic entity, the “Autosomal dominant non-syndromic hearing loss 20/26”. In these syndromes, typical craniofacial features, sensory impairment (vision and hearing) and intellectual disabilities are frequently present. Heart anomalies, renal and gastrointestinal involvement and seizure are also common. Wide inter- and intra-familial variety in the phenotypic spectrum is reported. Some phenotypic aspects of these syndromes are not yet fully described, such as the degree and progression of HL, and better knowledge of them could be useful for correct follow-up and treatment. **Methods and Results**: In this study, we report two cases of children with HL and diagnosis of BRWS and a review of the current literature on HL in these syndromes.

## 1. Introduction

BRWS is an autosomal dominant condition caused by pathogenic variants in the two genes ACTB and ACTG1 located on chromosome 7 (band numbered 7p22.1) and 17 (band numbered 17q25.3), respectively. The ACTB gene is involved in BRWS1 (MIM#243310) and the ACTG1 gene in BRWS2 (MIM# 614583). In addition, cerebrofrontofacial syndrome type 1 and Type 3, recently associated with heterozygous gain-of-function variants in one of the two genes, and Fryns–Aftimos syndrome, due to chromosomal deletions involving the ACTB gene, are now considered part of the BRWS syndromic spectrum. These disorders used to be distinguished by the prevalence and/or severity of some signs and symptoms. 

Both genes encode actins, cytoskeletal proteins that are found throughout the organism, and in the auditory system are predominant in the cytoskeleton of auditory hair cells, such as stereocilia, cuticular plate and adherens junctions. In particular, the ACTG1(MIM#102560) gene encodes for a gamma actin while the ACTB gene encodes for a beta (β)-actin [1,2,3]. To date, approximately 150 variants are described for the ACTB gene [4] and about 90 variants for the ACTG1 gene [5]. While pathogenic variants at the ACTB gene are associated only with the BRWS1 complex, there are two clinical situations associated with variants of the ACTG1 gene, one syndromic, i.e., BRWS2 (MIM#614583), and one non-syndromic with only auditory alterations, i.e., “Autosomal dominant non-syndromic sensorineural deafness 20/26 (DFNA20/26,MIM#604717)”. In DFNA20/26, the isolated progressive HL is often bilateral with initial involvement of the high frequencies (6000 and 8000 Hz) and secondary involvement of all other frequencies. HL occurs in the first to second decade of life with progression to profound HL by the 4th to 6th decade [6,7,8].

Both forms of BRWS are characterized by possible involvement of several organs and specific but not exclusive dysmorphisms. Facial dysmorphisms, such as hypertelorism, a broad nose with a large tip and a prominent root, high-arched eyebrows and retinal coloboma are often reported. Short stature, eye abnormalities (micro cornea, macrophthalmia, iris or retinal coloboma), microcephaly, muscle involvement and congenital anomalies of the brain can also be present. In some cases, intellectual disability, seizures, heart defects, renal and gastrointestinal malformations and cleft lip palate occur. The concomitant presence of sensorineural hearing loss (SNHL) has been described, but the characteristics of this deficit are poorly described [9,10,11,12].

Considering the overlap of signs or symptoms, differentiation of the forms of BRWS based on phenotype is often not possible.

In addition, the symptoms and signs present in BRW syndromes overlap with those of other syndromes, making a clinically based differential diagnosis difficult or often impossible. It is now accepted that only genetic testing can provide an accurate diagnosis. At present, some aspects of the above syndromes are not still under-reported and deserve further investigation. To date, the degree and the progression of HL in the different forms of BRWS are not yet well described. In this report we describe two cases of children with HL and diagnosis of BRWS and “Autosomal dominant non-syndromic hearing loss 20/26” to improve knowledge of the clinical features, especially audiological changes during the follow-up of these patients. Moreover, a narrative review of the literature was carried out to make a comparative assessment of the characteristics of hearing impairment (such as frequency, type, and progression) in our two cases and in those previously reported with the different variants of the ACTB and ACTG1 genes.

## 2. Case Reports

Informed consent was obtained from the parents of the subjects who were involved in the study.

**Figure 1:**
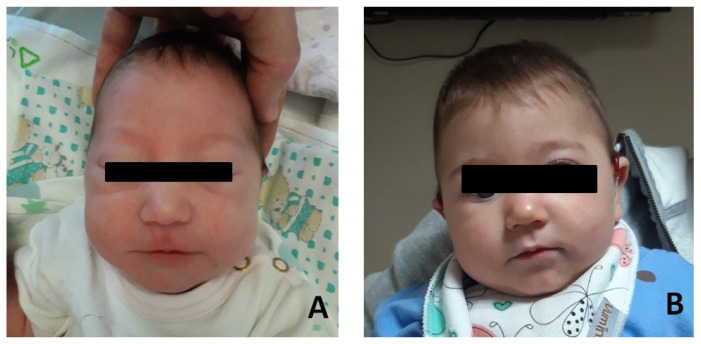
Photographs of the patient#1. (**A**) Patient at few months after birth. (**B**) Patient at age 12 months.

Patient #1: female, normal pregnancy, born in 2022 at full-term (38 + 6 weeks) by C-section. Due to respiratory distress, she was admitted to the neonatal intensive care unit. Respiratory support with CPAP was applied for 15 min. She underwent endoscopic examination with a diagnosis of laryngomalacia type I (Olney’s classification) (Olney DR), which resolved spontaneously. Dysmorphological examination revealed mild mandibular micrognathia, bitemporal narrowing with hypotelorism, elongated eyelid rims, well-shaped and angulated but inferiorly set ear pinnae (Figure 1). Abdominal and heart ultrasound ophthalmic examination were within normal limits. Brain MR (magnetic resonance) resulted normal, as were the VII or VIII cranial nerves.

Both parents had normal hearing and no family history of HL.

The newborn hearing screening test resulted in REFER bilaterally. She underwent ABR (Auditory Brainstem Response) at 40 days old: V wave was detected at 80 dB HL bilaterally. 

When she was 4 months old, she was referred to our audiology centre, where the hearing tests showed that

- ABR: V wave was detected at 60 dB on the left ear and 90 dB on the right ear;
- Impedenzometry: type A tympanogram bilaterally, acoustic reflex was present only on the left side.

She had a bilateral hearing aid (HA) since the age of 5 months. A control performed two months later (6 months old) showed:

- ABR: V wave was detected at 60 dB bilaterally;
- Distortion-product otoacoustic emission (DPOAE): partially present bilaterally;
- Visual reinforcement audiometry (VRA): PTA (pure tone average) of 65 dB without HA and 40 dB with HA.

**Figure 2:**
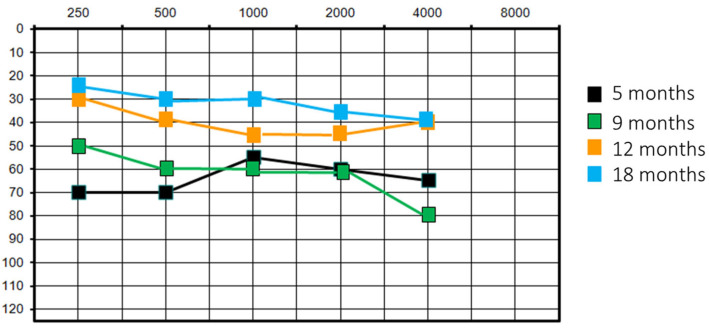
Patient #1 unaided pure tone audiometry at different follow-ups.

She had a re-evaluation at 9 months of age with similar results. At the 1-year follow-up, there was an improvement in audiological performance: PTA was at 40 dB on the left side and 35 dB on the right (VRA) and acoustic reflex was present bilaterally. VRA was also performed with HA, with overlapping results. This clinical finding was corroborated by the patient’s behaviour of no longer tolerating HA; perceptual–auditory skills were in accordance with chronological age. It was decided to suspend the use of HA. At the audiological control at 1.5 years old, PTA was 30 dB (VRA) and acoustic reflex was present bilaterally. However, the audiometric curve showed a downward trend on the high frequencies (40 dB threshold on 4000 Hz) (Figure 2).

A genetic analysis of the baby was conducted using karyotype and CGH array analysis: standard karyotype result was 46, XX normal female; CGH array analysis detected a microduplication of the long arm of chromosome 21 region 21q22.13 of approximately 69.94 kb (arr[GRCh37] 21q22.13(38404608_38474549)x3 mat) of maternal origin. This microduplication has been defined as being of uncertain significance (small duplication, reported in low frequency in the DGV, inherited from a healthy parent, involving only one OMIM morbid gene-PIGP with autosomic recessive inheritance and unrelated phenotype, no overlapping microduplication in clinical databases such as DECIPHER and ClinVar). Further analysis using exome sequencing of genes involved in HL showed a de novo heterozygous c.629G > A p. variant (Arg210His) in the ACTG1 gene classified as probably pathogenic. The variant is not reported in the GnomAD database; it is considered pathogenic by in silico predictors such as Polyphen2 and can be classified as likely pathogenic (class 4) according to ACMG guidelines (PS2 strong, PM1 moderate, PM2 moderate, PP3 supporting, PP5 supporting).

Considering that this patient presents only mild HL and facial dysmorphisms compatible with those of BRWS and that she has a pathogenetic variant in ACTG1, the diagnosis of “non-syndromic sensorineural hearing loss type 20/26” was made. This is a form of BRWS2 that expresses only HL or minimal other alterations.

**Figure 3:**
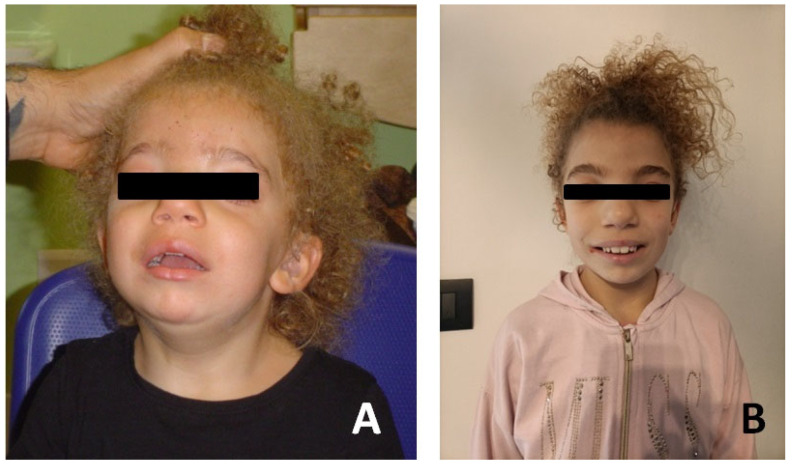
Photographs of the patient#2. (**A**) Patient at age of 18 months. (**B**) Patient at age 8 years.

Patient #2 is a female, born in 2014 at 36 + 2 weeks of normal pregnancy by natural childbirth. A standard karyotype and in utero MRI were performed at 22 weeks’ gestation for increased nuchal translucency on prenatal ultrasound with normal results. She had good cardio-respiratory adaptation at birth. Dysmorphological examination revealed some facial dysmorphisms: hypertelorism and telecanthus with downslanting palpebral fissures, ptosis of eyelids, arched eyebrows, nose with widened root and globular tip, prominent full and wide cheeks, large mouth, thin upper lip, thick lower lip and short neck (Figure 3).

She also showed other dysmorphisms, including: hypertrophic vaginal mucosa with extroflexions, hypotrophy of the buttocks without gluteal groove with evident and angulated sacrum and diffuse eczematous lesions of the skin. An abdominal ultrasound showed mild ectasia of upper colico-pyelic cavities and II degree hydronephrosis on the left. Right kidney revealed a discrete dilatation of renal pelvis and of colico-pyelic cavities suggestive of a mild nephrosis. Perimembranous ventricular septal defect (with mild left-to-right atrial shunt) was diagnosed on cardiac ultrasound. Cerebral MRI reported alteration of Virchow–Robin perivascular spaces in bilateral peritrigonal region (finding within standard limits). Ophthalmic examination and electroencephalography were within normal limits. She had a psychomotor development delay and a hearing problem. Both parents had normal hearing and there was no family history of HL. The newborn hearing screening, performed with OAE test, resulted pass bilaterally. Due to parental suspicion of deafness, the child underwent audiological evaluation with ABR at the age of 15 months: V wave was detected at 90 dB HL bilaterally. Consequently, bilateral HA was prescribed. Subsequent audiological evaluation yielded the following results:

- Otoscopic examination: bilateral otitis media with effusion;
- Audiological test: mean PTA was 100 dB HL on the right ear and 90 dB HL on the left ear without HA; mean PTA was 55 dB with bilateral HA;
- Impedenzometry: type B tympanogram bilaterally; ABR: V wave was detected at 70 dB on the left ear and was absent on the right ear;
- Speech evaluation: poor perceptual-auditory skills, only inconstant voice detection. Language development was at the pre-verbal stage. A psychomotor delay was present.

**Figure 4:**
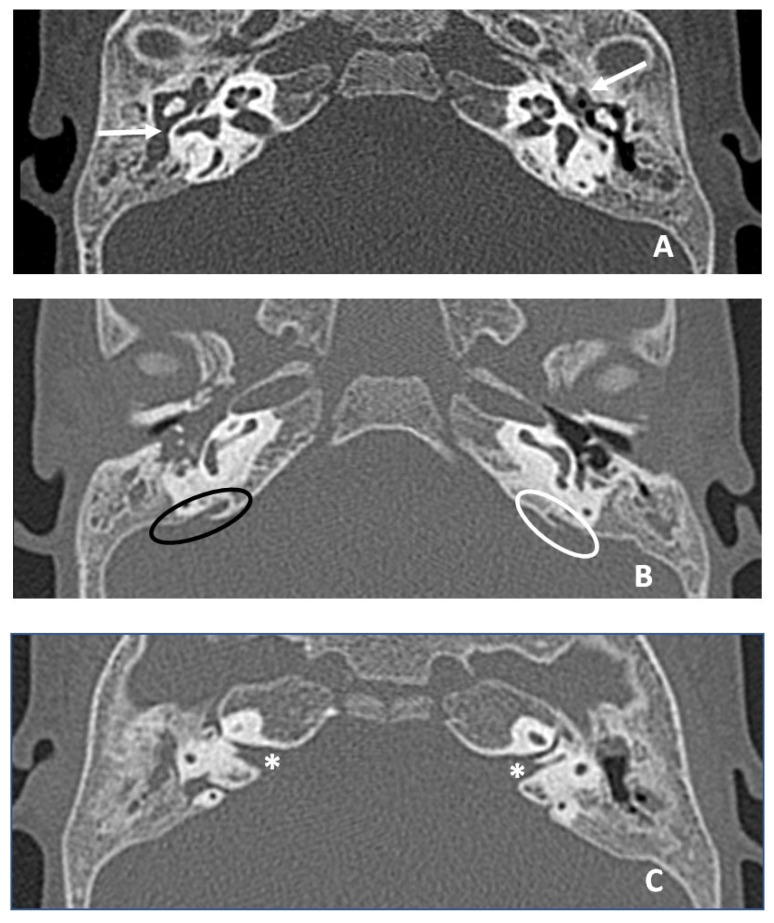
CT images in axial plane. (**A**) White arrows indicate hypodense material in middle ear and mastoid compatible with otitis media with effusion, greater on the right. (**B**) Ovals indicate the vestibular aqueduct bilaterally, black oval on the right (slightly enlarged aqueduct, larger than the diameter of posterior semicircular canal) and white oval on the left. (**C**) White asterisks indicate Bill bar bilaterally, thickened and more developed in length than normal.

The patient underwent an ear CT (computed tomography) scan, which showed bilateral endotimpanic effusion with right tympanic cavity and mastoid cells occupied by hypodense/fluid material. The presence of fluid material was also detected in the epi- and mesotympanic spaces on the left ear. This finding was compatible with adenoid hypertrophy. The ossicular chain was regular bilaterally, the oval and round windows were pervious bilaterally; cochlear and vestibular structures were regularly conformed and the course of the facial nerve was normal. The vestibular aqueduct was mildly enlarged on the right ear. A thickened and elongated Bill bar was noted bilaterally (Figure 4).

In view of the severity of the hearing loss, a right cochlear implant (CI) was indicated to optimise future rehabilitation. At the age of 2, she was implanted with a Medel Flex 28 CI model. The patient’s abilities gradually improved in subsequent follow-ups. At the last evaluation (8 year after CI surgery) the tests showed:

- Pure tone audiometry without hearing device: hearing residuals on the right, mean PTA of 75 dB on the left;
- Speech perception without hearing device: 100% of intelligibility at 80 dB on the left, only detection threshold at 90 dB on the right;
- Pure tone audiometry with right CI and left HA: mean free field PTA equal to 40 dB;
- Speech perception with right CI and left HA: 100% of intelligibility at 50 dB;
- Speech evaluation: phonetic skills were evolved with increased inventory. Poor vocal quality, presence of phonological processes of simplification of phonological structure and intelligibility of spontaneous production remains poor.

The patient was referred to a genetic examination and a missense variant c.1043C > T (p.Ser348lEU) was found in the ACTB gene compatible with BRWS1 diagnosis. This variant was found in heterozygosity and found to be de novo, being absent in her parents. The variant is not reported in the GnomAD database; it is considered pathogenic by in silico predictors such as Polyphen2 and Mutation taster and can be classified as likely pathogenic (class 4) according to ACMG guidelines (PS2 strong, PM1 moderate, PM2 moderate, PP2 supporting, PP3 supporting, PP5 supporting).

## 3. Literature Search

In order to better understand the characteristics of the HL loss in BWS cases, such as age at diagnosis, frequency, typology and progression of the HL, in addition to the two cases now described, we have considered those previously reported in the literature. For this purpose, we conducted a narrative review of relevant papers on patients with pathogenic variants of the ACTB and ACTG1 genes.

The authors searched the Pubmed and Cochrane Library databases for relevant papers. Two separate searches were performed, one using “ACTB gene” and the other using “ACTG1 gene” as keywords. For both, peer-reviewed research articles and case reports were included from inception until the time of conducting the research. The references extracted from the first step were then subjected to a secondary search. Only full-length original communications and peer-reviewed research articles and case reports were accepted. The search was limited to human subjects and English-language publications. Articles were reviewed by title and abstract or potential relevance to this topic. In addition, if the title and/or abstract did not clearly indicate its degree of relevance, then the article itself was reviewed. The Population, Intervention, Control, Outcomes, and Study design (PICOS) strategy [13] was used to draft criteria for inclusion and exclusion as precisely as possible. To be included in the review, studies had to meet the following PICOS criteria: (1) population: patients affected by ACTB or ACTG1 variants; (2) intervention: description of clinical characteristics in subjects with ACTB or ACTG1 variants; (3) control: subject without ACTB and ACTG1 variants or studies/subjects without clinical description; (4) outcomes: presence of HL; and (5) study design: clinical studies with clinical characteristic description. Articles published in peer-reviewed journals in the English language were included. Studies describing editorial letters, legal cases, interviews, discussion papers, clinical protocols, or presentations were not included. Identified articles were assessed for relevance by examining titles and abstracts.

**Figure 5:**
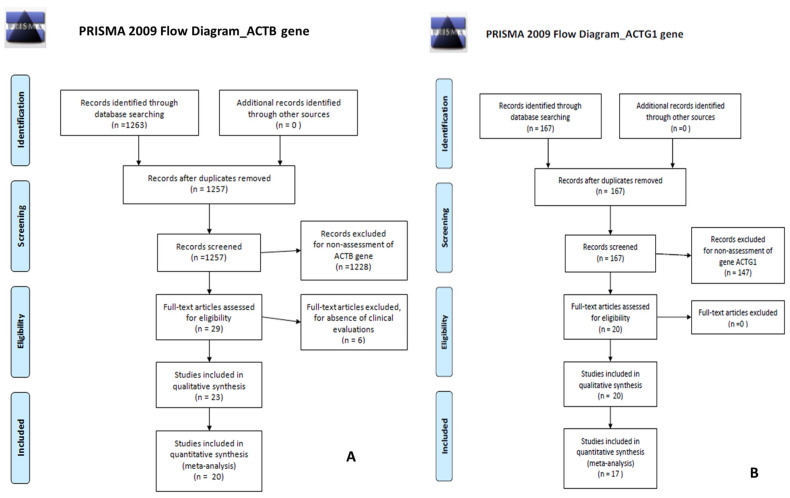
(**A**) PRISMA flow-charts for ACTB gene; (**B**) PRISMA flow-charts for ACTG1 gene.

The search for “ACTB gene” found 1263 papers of which 1249 were in PubMed and 14 in the Cochrane Library. The search for “ACTG1 gene” found 167 papers, of which 167 were in PubMed and 0 in the Cochrane Library. The PRISMA [14] flow-charts in Figure 5A (for ACTB gene) and Figure 5B (for ACTG1 gene) illustrate details of the searches and selection procedure including the number of duplicates removed, the number of articles that were excluded, and the reasons for their exclusion. 

For the research concerning the ACTB gene, after removing duplicates, 1257 references remained. After screening the abstracts and titles of these 1257 articles, a further 1228 articles were excluded. After selection, cross-reference checking, and review for clinical relevance, 20 studies met the inclusion criteria for this review. 

For the research concerning the ACG1 gene, we found no duplicates, and 167 references were evaluated. After screening the abstracts and titles of these 167 articles, a further 147 articles were excluded. After selection, cross-reference checking, and review for clinical relevance, 17 studies met the inclusion criteria for this review.

## 4. Hearing Loss in Baraitser–Winter Syndrome: Evidence Synthesis

The audiological and clinical characteristics of subjects affected by pathogenic variants of the ACTB gene are presented in Table A1 in Appendix A.

To date, 19 variant-related phenotypes are described. The most frequently encountered variant is c.547C > T (p.Arg183Trp), reported in six clinical studies [15,16,17,18,19,20]. This variant and the variants c.484A> G (p.Thr162Ala) [20], c.586C > T (p.Arg196Cys) (in one out of two case reports) [12,21] and c.826G > A (p.Glu276Lys) [22], are associated with a hearing impairment. Age of onset, time course of HL and use of HA are only described in cases with the c.547C > T variant. In this variant, the diagnosis of the HL is made at birth or during the first few months of life, and a progression of the hearing loss over time is described in two of the six cases. The use of hearing aids for auditory rehabilitation (specifically, a CI) is only reported in the paper by Skogseid et al. [15]. Finally, in most patients with the c.547C > T variant, the presence of dystonia, facial dysmorphisms, motor and language delays, renal and genital anomalies and, in only a few cases, motor and step acquisition delay, epilepsy and mild intellectual disability are described [15,16,17,18,19,20]. Audiometric tests are reported in the cases with the variants c.220 G > A (p.Gly74Ser), c.359C4T (p.Thr120Ile) and c.617G > A (p.Arg206Gl), but no hearing impairment was found [12,23. No information on hearing ability or whether it was assessed was reported in any of the remaining clinical reports.

Table A2 in Appendix A shows the clinical characteristics related to the variants of the ACTG1 gene. Hearing impairment has been described in almost all variants. The study by Chacon-Camacho et al. [23], describing the variants c.176A > G, p.Gln59Arg and c.608C > T, p.Thr203Met in two patients, is the only one that does not report any hearing impairment or investigate hearing ability. The age of onset of hearing impairment varies from birth to the third decade of life. In most cases, the diagnosis was made in the first or second decade of life. Only in the study by Miyajima et al. [24] is the onset of hearing impairment between the 4th and 6th decade of life in individuals with the c.895 C > G (p.L299V) and c.994 C > T (p.P332S) variants reported. The progression of the hearing deficit over time is reported to be progressive, with the exception of one case described as stable (c.142 G > C p.G48R) and another reporting an improvement in audiometric threshold over time (c.542C > T, p.Ala181Val). The type of device used was not described in all cases, but the majority of subjects used HA or CI. Given the progressiveness of the hearing deficit, the use of HA followed by CI was described in two cases [25]. In one subject, an electroacoustic stimulation (EAS) was used to adapt the HL threshold to a profound hearing impairment at high frequencies and a minor deficit at low frequencies [24].

Most variants in the ACTG1 gene have isolated HL. In syndromic cases, the other reported alterations are cleft-lip/palate in the variant c.94C > T, dysmorphic features in the variants c.617G > A p.(Arg206Gln),c.1003C > T; p.(Arg335Cys), eye problems in the variants c.625G > A (p. Val209Met) and p.Ala58Val, cardiac abnormalities in c.617G > A p.(Arg206Gln) and c.1003C > T; p.(Arg335Cys) variants and psychomotor delay associated with microcephaly and short stature in the c.617G > A p.(Arg206Gln) variant [9,25,26,27,28]. In the work by Chacon-Camacho et al., clinical features such as eye malformations, dysmorphic facial features, and psychomotor delay in the variant c.176A > G, p.Gln59Arg and eyes and genital anomalies, facial dysmorphism, psychomotor delay and encephalic alterations at MRI in c.608C > T, p.Thr203Met variant are described [29]. This study does not report whether auditory abilities were assessed. The only case report describing improvement in hearing over time describes concomitant renal, genital, and cardiac involvement, encephalic changes, and dysmorphic features [30].

## 5. Discussion

The literature reports that the different types of BRWS syndrome are characterised by the variable presence of facial dysmorphisms, changes in various organs and apparatus, and concomitant hearing impairment [11,31].

With regard to HL, characteristics such as age of onset, degree of HL, possible variability over time and the co-occurrence of other signs or symptoms are poorly described. 

In this paper, we have analysed two cases characterised by hearing impairment in two different types of the BRWS syndrome: “Syndromic sensorineural hearing loss type 20/26”, a BRWS Type 2 with the presence of isolated hearing impairment (patient# 1), and one with Baraitser–Winter syndrome type 1 (patient#2). 

Investigations performed on patient #1 revealed the presence of a heterozygous variant (c.629G > A p; Arg210His) of the ACTG1 gene, with de novo onset, classified as “probably pathogenic”. The HL in this young patient was compared with other cases in a narrative review of the literature on phenotypic features in variants of the same gene. The review did not find any studies or case reports reporting the c.629G > A p variant. This variant is present in the ClinVar clinical database in three cases, interpreted as follows: pathogenic, likely pathogenic and uncertain significance [32]. HL is not reported in any of the three cases. In contrast, our systematic review found that HL is very common in ACTG1 variants. In fact, 23 variants were found (reported in 16 articles) out of the 25 in which the defect was described. Only one article, reporting on two cases with two different variants, reported no hearing symptoms [23]. It has been reported that HL is progressive in the majority of cases. In contrast, our patient #1 showed an improvement in his hearing threshold over time until he had normal mid-frequency thresholds at the age of 1.5 years. There is only one case report in which an improvement in the hearing threshold has been described [30]. The authors attributed this improvement to the resolution of an initial concomitant catarrhal component of the middle ear that was present at the time of the first evaluation. In contrast to this study, our patient did not have an endotympanic effusion component (objectified by the presence of a bilateral type A tympanogram). For these reasons, it can be said that our patient has experienced an improvement in her hearing threshold over the course of time. This improvement is described in the literature as possible, especially given the presence of pre- and perinatal risk factors for HL (especially in premature infants) [33]. In children without risk factors for HL, an improvement in hearing threshold has also been described in the early years [34]. Despite the improvement in the mid to low frequencies, Patient #1 still has a slight hearing deficit in the high frequencies. This deficit is thought to be related to the presence of the ACTG1 gene variant, as several papers report an initial hearing impairment in the early years of life, limited to high frequencies [35,36]. For these reasons, it can be assumed that the improvement in our patient’s hearing threshold is not due to the ACTG1 gene variant, but that the persistence of the high-frequency deficit is due to the variant itself. In addition, given the genetic profile, a progressive hearing deficit over time cannot be excluded, with possible onset from the 2nd to 3rd decade of life. It will also be interesting to assess other audiological aspects over time. In fact, tinnitus and episodes of vertigo and/or dizziness have been reported in the literature in up to 50% of cases of patients with variants in the ACTG1 gene [24].

Analysis of the ACTB gene found fewer papers reporting hearing impairment than analysis of the ACTG1 gene. In agreement with our results, Riviere et al. report that hearing impairment is present in 50% of cases described for the ACTB gene and 83.3% of cases for the ACTG1 gene [10]. Our narrative review found that the p.Ser348Leu variant (c.1043C > T) in the ACTB gene, present in our patient#2, was previously described in twins in a paper by Sibbin et al. [37]. The authors report the presence of both craniofacial features and gastrointestinal malformations (atresia). A child showed pulmonary hypoplasia and died at 1 h of age. The second twin presented laryngeal dysgenesis, trachea malformations, cardiac and hematologic defects and she also died, on day 23. None of the clinical features present in the twins were found in our patient#2. There is also no mention of hearing in Sabbin’s work. The short lifespans of the two small ones may explain the lack of such data. The p.Ser348Leu variant (c.1043C > T) of our patient #2 is also reported in the ClinVar clinical database in seven cases described as pathogenic (4); likely pathogenic (2); uncertain significance (1). HL is not reported in any of the seven cases [38].

The hearing characteristics of our patient #2 were in line with what was found in the literature review for the ACTB gene. In fact, she had a diagnosis of HL at birth. Consistently, in seven of the nine cases reviewed, hearing impairment was diagnosed by the age of 2 years. On the contrary, it is difficult to extrapolate information on the progressiveness or use of hearing aids from the information obtained from the review. One possible bias is that in most cases there is a profound deficit at birth, which is not characterised by progressivity because it is already extreme. From the analysis of the ACTB identified, the review showed some common clinical characteristics, such as facial dysmorphisms, motor and developmental delay, dystonia, cleft lip and palate, ocular, cardiac, gastrointestinal and renal abnormalities. Four papers also reported the presence of cerebral MRI abnormalities [12,21,39,40,41]. Specifically, features such as hypoplastic corpus callosum [12,21,40], pachygyria, polymicrogyria and trigonocephaly [12,21,40], grey matter alterations [39,40] and dysmorphic ventricles [39] are described. These brain MRI changes were not found in our patient #2, who reported alteration of Virchow–Robin perivascular spaces in the bilateral peritrigonal region. Additionally, the CT exam showed a mildly enlarged right vestibular aqueduct and the presence of a bilaterally thickened and elongated Bill bar in the internal auditory canal. The presence of the vestibular aqueduct on the right side may have a correlation with HL on that side and also with possible deterioration over time. It has been reported in the literature that the presence of this anatomical variant is associated with a deterioration over time to a profound degree of HL [42]. In our patient, passing the hearing screening at birth may indicate a good hearing threshold in early life, and the presence of the enlarged vestibular aqueduct may be associated with the observed hearing deterioration. More controversial is the aetiology of left-sided HL in the absence of ear malformation. In contrast to the vestibular aqueduct, the presence of the Bill bar is not associated with a hearing impairment, because it is only an anatomical structure that divides the superior part of the internal auditory canal into an anterior and posterior part containing the facial nerve and the superior vestibular nerve, respectively.

## 6. Conclusions

This paper shows that there is a lack of knowledge about some aspects of the auditory phenotype in patients with Baraitser–Winter syndrome. The review of the literature on the clinical features of the pathogenic variants of the ACTB and ACTG1 genes shows a significantly higher incidence of hearing impairment in ACTG1 gene variants than in those of the ACTB gene. HL associated with the ACTG1 gene tends to be early onset (between birth and the third decade of life) and progressive, requiring the use of HA or CI in the most severe cases. Our case, on the contrary, shows an improvement in the post-diagnosis period, suggesting the need for comprehensive and serious audiological controls in individuals with ACTG1 gene variants. In patients with variants of the ACTB gene, due to the scarce literature on the HL cases, it can only be assumed that its possible onset in the early years of life and a subsequent progressive course exists. It is therefore necessary to better study and describe hearing ability and its time course in patients with variants for this gene.

## Data Availability

The data presented in this study are available on request from the corresponding author.

## Appendix A

**Table A1:** Audiological and clinical characteristics in pathogenic variants of the ACTB gene.

Gene ACTB									
	Change cDNA Amino Acid Change	N° Patients	Gender	HL	Age Onset HL	Progression	Hearing Intervention	Other Malformations	Reference
1	c.208C > G, p.Pro70Ala	1	Male	Not reported	N/A	N/A	N/A	Craniofacial features, intellectual disability, motor and speech delay, eye problems	[43]
2	c.220 G4A p.Gly74Ser	1	Female	No	N/A	N/A	N/A	Heart defect, cleft lip and palate, motor delay, facial dysmorphism	[12]
3	c.359C4T p.Thr120Ile	1	Female	No	N/A	N/A	N/A	Eye problems, encephalic alterations, gastro-intestinal, renal alterations, facial dysmorphism, epilepsy, motor delay	[12]
4	c.478A > G (p.Thr160Ala)	1	Male	Not reported	N/A	N/A	N/A	Facial features, eye problems thrombocytopenia, intellectual developmental delay and attention deficit	[44]
5	c.484A > G, p.Thr162Ala	1	Male	Yes	Not reported	Not reported	Not reported	Development retardation, psycho-motor retardation, hypotonia, cerebral malformations, epilepsy.	[21]
6	c.511C > T, p.Leu171Phe	1	Female	Not reported	N/A	N/A	N/A	Cleft soft palate, ventricular arrhythmia, thrombocytopenia, dysmorphic features, development delay, eye problems	[43]
7	c.547C > T p.Arg183Trp	1	Male	Yes	2.5 years	Not reported	CI	Facial dysmorphism	[15]
		1	Male	Yes	8 months	Not reported	Not reported	Dystonia, motor and language delays, mild intellectual disability	[16]
		1	Male	Yes	Birth	Not reported	Not reported	Dystonia	[17]
		1	Female	Yes	Congenital	Progressive	N/A	Facial dysmorphism, motor delay	[18]
		1	Female	Yes	Birth	Not reported	Not reported	Dystonia Craniofacial dysmorphism	[19]
		1	Female	Yes	Childhood	Progressive		Dystonia, epilepsy, scoliosis	[20]
8	c.586C > T (p.Arg196Cys)	1	Female	Not reported	N/A	N/A	N/A	Renal problem, facial dysmorphism, limb problem, heart anomalies, cerebral MRI alterations	[40]
		1	Male	Yes	Not reported	Not reported	Not reported	Craniofacial anomalies, renal and genital anomalies	[12]
9	c.617G > A, p.Arg206Gl	2 (one family)	1 male 1 female	No	N/A	N/A	N/A	Intellectual disability, dysmorphic features, delayed motor and speech development, low muscle tone.	[29]
10	c.802G > T p.Gly268Arg	1	Male	Not reported	N/A	N/A	N/A	Hydrocephalus, cleft and lip palate, renal problems, dysmorphisms	[45]
11	c.826G > A (p.Glu276Lys)	1	Female	Yes	2	Not reported	Tube insertion	Facial features, brain abnormalities, ocular coloboma, cardiac defects, intellectual disabilities, short stature, developmental delay	[39]
12	c.938T > G p.(Met313Arg)	2	Males	Not reported	N/A	N/A	N/A	(1) Mild developmental delay, cleft lip, heart defect, microcephaly, leukocytosis and thrombocytopenia (2) Thrombocytopenia	[41]
13	c.992_1008del, p.(Ala331Val_fs*27)	2	1 Male 1 Female	Not reported	N/A	N/A	N/A	(1) Speech delay, borderline intellectual impairment and microcephaly, pseudarthrosis, facial anomalies (2) Mild intellectual disability, microcephaly, thrombocytopenia, facial anomalies	[41]
14	c.1012_1023del p.(Ser338_Ile341del)	1	Male	Not reported	N/A	N/A	N/A	Microcephaly, facial anomalies, speech delays, haematological anomalies	[41]
15	c.1043C > T; p.Ser348Leu	2 (one family, twin)	2 females	Not reported	N/A	N/A	N/A	(1) Craniofacial features, bowel atresia, pulmonary hypoplasia She died at 1 h of age (2) Craniofacial features, laryngeal dysgenesis, malformed trachea, jejunal atresia, megakaryocytes, hyperglycaemia, patent ductus arteriosus, ventricular septal defect. She died on day 23	[37]
16	c.1090G > A p.(Glu364Lys)	1	Female	Not reported	N/A	N/A	N/A	Developmental delay, microcephaly, thrombocytopenia, encephalic MRI alterations	[41]
17	c.a092_1105dup14 p.Ile369SerfsX18	1	Female	Not reported	N/A	N/A	N/A	Short stature, developmental delay, eye alterations, minor facial dysmorphism. motor and speech delay	[46]
18	c.1101dup p.(Ser368Leu_fs*13)	1	Female	Not reported	N/A	N/A	N/A	Photo-sensitivity, stomatitis, keratoconjunctivitis, thrombocytopenia, leukopenia, recurrent otitis media, furunculosis, polyarthralgia, intellectual impairment, short stature	[47]
19	c.1117A > T; p.Lys373Ter	5	3 male 2 female	Not reported	N/A	N/A	N/A	Grown retardation, speech and motor delay, heart problem, scoliosis, renal and genital anomalies	[48]

N/A: Not Applicable; CI: Cochlear Implant.

**Table A2:** Audiological and clinical characteristics in pathogenic variants of the ACG1 gene.

Gene ACTG1									
	Change	N° Patients	Gender	HL	Age Onset HL (Years)	Progression	Hearing Intervention	Other Malformations	Reference
1	c.94C > T	2 (one family)	Male	Yes	Birth	Unknown	CI	Cleft-lip palate	[49]
			Male	Yes	Prelingual	Unknown	None	No	[49]
		1	Male	Yes	3	Unknown	Unknown	No	[50]
2	c.102 C > G p.I34M	2 (one family)	Male	Yes	18	Progressive	HA	Not reported	[24]
			Female	Yes	15	Progressive	None	Not reported	[24]
3	c.110 G > A p.R37H	2 (one family)	Female	Yes	Unknown	Unknown	None	Not reported	[24]
			Male	Yes	10	Progressive	Unknown	Not reported	[24]
4	c.142 G > C p.G48R	4 (one family)	Female	Yes	11	Progressive	HA	Not reported	[24]
			Female	Yes	Not reported	Progressive	None	Not reported	[24]
			Male	Yes	7	Progressive	None	Not reported	[24]
			Female	Yes	Precritical	Unknown	None	Not reported	[24]
		4 (one family)	Female	Yes	11	Progressive	Not reported	Not reported	[35]
			Male	Yes	6	Stable	Not reported	Not reported	[35]
			Female	No	Not reported	Not reported	Not reported	Not reported	[35]
			Female	Yes	Not reported	Not reported	Not reported	Not reported	[35]
5	c.151G > A	8 (one family)		Yes	From birth to 34 years	5 of 8 progressive	HA (3 in selection for CI)	Not reported	[51]
6	c.176A > G, p.Gln59Arg	1	Male	Not reported	Not reported	Not reported	Not reported	Ptosis, coloboma, dysmorphic facial features, psychomotor delay, pachygyria	[23]
7	c.246 G > A p.M82I	1	Male	Yes	Unknown	Unknown	None	Not reported	[24]
8	c.266 C > T p.T89I	1	Male	Yes	Unknown	Progressive	HA	Not reported	[24]
9	c.353 A > T p.K118M	3 (2 families)	Female	Yes	26	Progressive	HA	Not reported	[24]
			Male	Yes	17	Progressive	HA	Not reported	[24]
			Male	Yes	17	Progressive	HA	Not reported	[24]
		2 (one family)	Male	Yes	17	Progressive	Not reported	Not reported	[35]
			Male	Yes	17	Not reported	Not reported	Not reported	[35]
		1	Female	Yes	26	Progressive	HA	Not reported	[35]
10	c.364A > G; p.I122V	11 (one family)	8 males, 3 females	Yes	Mean 6 years	Progressive	Not reported	No	[52]
11	c.493 A > G p.I165V	1	Male	Yes	11	Progressive	HA	Not reported	[24]
12	c.542C > T (NM_001614.5), p.Ala181Val (NP_001605.1)	1	Male	Yes	4 months	Hearing improving	HA (subsequently abandoned)	Renal, genital, cardiological, encephalic dysmorphic features	[30]
13	c.608C > T, p.Thr203Met	1	Male	Not reported	N/A	N/A	N/A	Strabismus, coloboma, facial dysmorphism, genital alterations, psychomotor delay, MRI: cortical dysplasia, pachygyria, short and thick corpus callosum with rostral agenesis, hypoplastic cerebellar vermis	[23]
14	c.617G > A p.(Arg206Gln)	1	Female	Yes	4	Progressive	Not reported	Psychomotor delay, microcephaly dysmorphic features, cardiac septal hypertrophy, abdominal swelling	[25]
15	c.625G > A (p. Val209Met)	1	Male	Yes	6	Progressive	Not reported	Myopia, astigmatism	[9]
16	c.638A > G (p.K213R)	8 (one family)	6 males 2 females	Yes	Second decade (7 cases) Birth (one case)	Progressive	Not reported	No	[53]
17	c.721 G > A p.E241K	3 (one family)	Female	Yes	14	Progressive	HA	Not reported	[24]
			Male	Yes	3	Progressive	HA	Not reported	[24]
			Female	Yes	3	Unknown	None	Not reported	[24]
		3 (one family)	Female	Yes	14	Not reported	HA	Moyamoya disease	[35]
			Male	Yes	4 years, 11 months	Not reported	HA	Developmental disorder	[35]
			Female	Yes	3	Not reported	Not reported	Not reported	[35]
18	c.791 C > T p.P264L	1	Female	Yes	12	Progressive	Unknown	Not reported	[24]
19	c.823 C > T p.H275Y	1	Female	Yes	34	Progressive	Unknown	Not reported	[24]
20	c.895 C > G p.L299V	4 (one family)	Male	Yes	46	Progressive	HA	Not reported	[24]
			Male	Yes	6	Progressive	EAS	Not reported	[24]
			Male	Yes	15	Progressive	HA	Not reported	[24]
			Male	Yes	Precritical	Progressive	None	Not reported	[24]
		4 (one family)	Male	Yes	12	Progressive	EAS	Not reported	[35]
			Male	Yes	15	Progressive	Not reported	Not reported	[35]
			Male	Yes	Not reported	Not reported	Not reported	Not reported	[35]
			Male	Yes	Not reported	Not reported	Not reported	Not reported	[35]
21	c.914 T > C p.M305T	1	Male	Yes	6	Progressive	EAS	Not reported	[24]
		3 (one family)	Female	Yes	Postlingual (about 30)	Progressive	HA	No	[25]
			Male	Yes	Postlingual (about 30)	Progressive	HA→ CI	No	[25]
			Male	Yes	Postlingual (about 30)	Progressive	HA→ CI	No	[25]
22	c.994 C > T p.P332S	2 (one family)	Male	Yes	59	Progressive	HA	Not reported	[24]
			Male	Yes	38	Progressive	Unknown	Not reported	[24]
		1	Male	Yes	35	Progressive	HA	Not reported	[24]
23	c.1003C > T; p.(Arg335Cys	1	Male	Yes	6	Not reported	Not reported	Craniofacial dysmorphia signs, cardiac and ophthalmological alterations.	[27]
	c.1109T > C; p.V370A	19 (one family)	12 females 7 males	Yes	From 6 to 24	Progressive	HA and 3 CI	Not reported	[8]
24	p.Ala58Val	3 (one family)	Male	Yes	7	Not reported	HA	Eye problems	[28]
			Male	Yes	4	Not reported	Not reported	Eye problems	[28]
			Female	Yes	Early childhood	Not reported	HA	Eye problems	[28]
25	Not reported	4 families	Male	Yes	Not reported	Progressive (high frequencies)	Not reported	Not reported	[36]
			Not reported	Yes	Not reported	Progressive	Not reported	Not reported	
			Not reported	Yes	Not reported	Progressive	Not reported	Not reported	
			Not reported	Yes	Not reported	Progressive	Not reported	Not reported	

N/A: Not Applicable; CI: Cochlear Implant; HA: Hearing Aids.

## References

[1] Sonnemann K.J., Fitzsimons D.P., Patel J.R., Liu Y., Schneider M.F., Moss R.L., Ervasti J.M. (2006). Cytoplasmic gamma-actin is not required for skeletal muscle development but its absence leads to a progressive myopathy. Dev. Cell.

[2] Perrin B.J., Sonnemann K.J., Ervasti J.M. (2010). Beta-actin and gamma-actin are each dispensable for auditory hair cell development but required for Stereocilia maintenance. PLoS Genet..

[3] Drummond M.C., Belyantseva I.A., Friderici K.H., Friedman T.B. (2012). Actin in hair cells and hearing loss. Hear. Res..

[4] Clin Var. https://www.ncbi.nlm.nih.gov/clinvar/?term=ACTB+gene.

[5] Clin Var. https://www.ncbi.nlm.nih.gov/clinvar/?term=ACTG1+gene.

[6] Morell R.J., Friderici K.H., Wei S., Elfenbein J.L., Friedman T.B., Fisher R.A. (2000). A new locus for late-onset, progressive, hereditary hearing loss DFNA20 maps to 17q25. Genomics.

[7] DeWan A.T., Parrado A.R., Leal S.M. (2003). A second kindred linked to DFNA20 (17q25.3) reduces the genetic interval. Clin. Genet..

[8] Rendtorff N.D., Zhu M., Fagerheim T., Antal T.L., Jones M., Teslovich T.M., Gillanders E.M., Barmada M., Teig E., Trent J.M. (2006). A novel missense mutation in ACTG1 causes dominant deafness in a Norwegian DFNA20/26 family, but ACTG1 mutations are not frequent among families with hereditary hearing impairment. Europ. J. Hum. Genet..

[9] Sorrentino U., Piccolo C., Rigon C., Brasson V., Trevisson E., Boaretto F., Martini A., Cassina M. (2021). DFNA20/26 and Other ACTG1-Associated Phenotypes: A Case Report and Review of the Literature. Audiol. Res..

[10] Riviere J.B., van Bon B.W.M., Hoischen A., Kholmanskikh S.S., O’Roak B.J., Gilissen C., Gijsen S., Sullivan C.T., Christian S.L., Abdul-Rahman O.A. (2012). De novo mutations in the actin genes ACTB and ACTG1 cause Baraitser-Winter syndrome. Nat. Genet..

[11] Verloes A., Di Donato N., Masliah-Planchon J., Jongmans M., Abdul-Raman O.A., Albrecht B., Allanson J., Brunner H., Bertola D., Chassaing N. (2015). Baraitser-Winter cerebrofrontofacial syndrome: Delineation of the spectrum in 42 cases. Eur. J. Hum. Genet..

[12] Di Donato N., Rump A., Koenig R., Der Kaloustian V.M., Halal F., Sonntag K., Krause C., Hackmann K., Hahn G., Schrock E. (2014). Severe forms of Baraitser-Winter syndrome are caused by ACTB mutations rather than ACTG1 mutations. Eur. J. Hum. Genet.

[13] Armstrong E.C. (1999). The well-built clinical question: The key to finding the best evidence efficently. WMJ.

[14] Moher D., Liberati A., Tetzlaff J., Altman D.G., PRISMA Group (2009). Reprint–preferred reporting items for systematic reviews and meta-analyses: The PRISMA statement. Phys. Ther..

[15] Skogseid I.M., Røsby O., Konglund A., Connelly J.P., Nedregaard B., Jablonski G.E., Kvernmo N., Stray-Pedersen A., Glover J.C. (2018). Dystonia-deafness syndrome caused by ACTB p.Arg183Trp heterozygosity shows striatal dopaminergic dysfunction and response to pallidal stimulation. J. Neurodev. Disord..

[16] Conboy E., Vairo F., Waggoner D., Ober C., Das S., Dhamija R., Klee E.W., Pichurin P. (2017). Pathogenic Variant in *ACTB*, p.Arg183Trp, Causes Juvenile-Onset Dystonia, Hearing Loss, and Developmental Delay without Midline Malformation. Case Rep. Genet..

[17] Kola S., Kandadai R.M., Kashyap M., Deepak S., Prasad V., Alugolu R., Borgohain R. (2023). Dystonia Deafness Syndrome: A Rare Deep Brain Stimulation Responsive Dystonia. Ann. Indian Acad. Neurol..

[18] Straccia G., Reale C., Castellani M., Colangelo I., Orunesu E., Meoni S., Moro E., Krack P., Prokisch H., Zech M. (2022). ACTB gene mutation in combined Dystonia-Deafness syndrome with parkinsonism: Expanding the phenotype and highlighting the long-term GPi DBS outcome. Park. Relat. Disord..

[19] Zavala L., Ziegler G., Morón D.G., Garretto N. (2021). Dystonia-Deafness Syndrome: *ACTB* Pathogenic Variant in an Argentinean Family. Mov. Disord. Clin. Pract..

[20] Freitas J.L., Vale T.C., Barsottini O.G.P., Pedroso J.L. (2019). Expanding the Phenotype of Dystonia-Deafness Syndrome Caused by *ACTB* Gene Mutation. Mov. Disord. Clin. Pract..

[21] Sun Y., Shen X., Li Q., Kong Q. (2018). Child with cerebral malformations and epilepsy. Int. J. Neurosci..

[22] Der Kaloustiana V.M., Pelletiera M., Costab T., Blackstonc D.R., Oudjhane K. (2001). A new syndrome with craniofacial and skeletal dysmorphisms and developmental delay. Clin. Dysmorphol..

[23] Chacon-Camacho O.F., Barragán-Arévalo T., Villarroel C.E., Almanza-Monterrubio M., Zenteno J.C. (2020). Previously undescribed phenotypic findings and novel ACTG1 gene pathogenic variants in Baraitser-Winter cerebrofrontofacial syndrome. Eur. J. Med. Genet..

[24] Miyajima H., Moteki H., Day T., Nishio S.Y., Murata T., Ikezono T., Takeda H., Abe S., Iwasaki S., Takahashi M. (2020). Novel ACTG1 mutations in patients identified by massively parallel DNA sequencing cause progressive hearing loss. Sci. Rep..

[25] Park G., Gim J., Kim A.R., Han K.H., Kim H.S., Oh S.H., Park T., Park W.Y., Choi B.Y. (2013). Multiphasic analysis of whole exome sequencing data identifies a novel mutation of ACTG1 in a nonsyndromic hearing loss family. BMC Genom..

[26] Graziani L., Cinnirella G., Ferradini V., Conte C., Bascio F.L., Bengala M., Sangiuolo F., Novelli G. (2023). A likely pathogenic ACTG1 variant in a child showing partial phenotypic overlap with Baraitser-Winter syndrome. Am. J. Med. Genet. A.

[27] Göbe T., Berninger L., Schlump A., Feige B., Nickel K.R., Schiele M.A., van Elst L.T., Hotz A., Alter S., Domschke K. (2022). Obsessive–compulsive symptoms in ACTG1 associated Baraitser Winter cerebrofrontofacial syndrome. J. Neural. Transm..

[28] Kemerley A., Sloan C., Pfeifer W., Smith R., Drack A. (2017). A novel mutation in ACTG1 causing Baraitser-Winter syndrome with extremely variable expressivity in three generations. Ophthalmic Genet..

[29] Hampshire K., Martin P.M., Carlston C., Slavotinek A. (2020). Baraitser-Winter cerebrofrontofacial syndrome: Report of two adult siblings. Am. J. Med. Genet. A.

[30] Dawidziuk M., Kutkowska-Kazmierczak A., Bukowska-Olech E., Jurek M., Kalka E., Guilbride D.L., Furmanek M.I., Bekiesinska-Figatowska M., Bal J., Gawlinski P. (2022). De Novo ACTG1 Variant Expands the Phenotype and Genotype of Partial Deafness and Baraitser-Winter Syndrome. Int. J. Mol. Sci..

[31] Yates T.M., Turner C.L., Firth H.V., Berg J., Pilz D.T. (2017). Baraitser-Winter cerebrofrontofacial syndrome. Clin. Genet..

[32] National Center for Biotechnology Information ClinVar; [VCV000449191.7]. https://www.ncbi.nlm.nih.gov/clinvar/variation/VCV000449191.7.

[33] Aldè M., Di Berardino F., Ambrosetti U., Barozzi S., Piatti G., Consonni D., Zanetti D., Pignataro L., Cantarella G. (2022). Hearing outcomes in preterm infants with confirmed hearing loss. Int. J. Pediatr. Otorhinolaryngol..

[34] Bovo R., Trevisi P., Ghiselli S., Benatti A., Martini A. (2015). Is very early hearing assessment always reliable in selecting patients for cochlear implants? A case series study. Int. J. Pediatr. Otorhinolaryngol..

[35] Miyagawa M., Nishio S.A., Ichinose A., Iwasaki S., Murata T., Kitajiri S.I., Usami S.I. (2015). Mutational spectrum and clinical features of patients with ACTG1 mutations identified by massively parallel DNA sequencing. Ann. Otol. Rhinol. Laryngol..

[36] Zhu M., Yang T., Wei S., DeWan A.T., Morell R.J., Elfenbein J.L., Fisher R.A., Leal S.M., Smith R.J., Friderici K.H. (2003). Mutations in the gamma-actin gene (ACTG1) are associated with dominant progressive deafness (DFNA20/26). Am. J. Hum. Genet..

[37] Sibbin K., Yap P., Nyaga D., Heller R., Evans S., Strachan K., Alburaiky S., Nguyen H.M.A., Hermann-Le Denmat S., Ganley A.R.D. (2022). A de novo ACTB gene pathogenic variant in identical twins with phenotypic variation for hydrops and jejunal atresia. Am. J. Med. Genet. A.

[38] National Center for Biotechnology Information ClinVar; [VCV000279997.28]. https://www.ncbi.nlm.nih.gov/clinvar/variation/VCV000279997.28.

[39] Choi G.J., Kim M.S., Park H., Kim J.Y., Choi J.M., Lee S.M., Jang J.H., Cho S.Y., Jin D.K. (2020). The First Korean Case of Baraitser-Winter Cerebro-Fronto-Facial Syndrome with a Novel Mutation in *ACTB* Diagnosed Via Targeted Gene Panel Sequencing and Literature Review. Ann. Clin. Lab. Sci..

[40] Eker H.K., Derinkuyu B.E., Ünal S., Masliah-Planchon J., Drunat S., Verloes A. (2014). Cerebro-fronto-facial syndrome type 3 with polymicrogyria: A clinical presentation of Baraitser-Winter syndrome. Eur. J. Med. Genet..

[41] Latham S.L., Ehmke N., Reinke P.Y.A., Taft M.H., Eicke D., Reindl T., Stenzel W., Lyons M.J., Friez M.J., Lee J.A. (2018). Variants in exons 5 and 6 of ACTB cause syndromic thrombocytopenia. Nat. Commun..

[42] Ruthberg J.S., Kocharyan A., Farrokhian N., Stahl M.C., Hicks K., Scarborough J., Murray G.S., Wu S., Manzoor N., Otteson T. (2022). Hearing loss patterns in enlarged vestibular aqueduct syndrome: Do fluctuations have clinical significance?. Int. J. Pediatr. Otorhinolaryngol..

[43] Sandestig A., Green A., Jonasson J., Vogt H., Wahlström J., Pepler A., Ellnebo K., Biskup S., Stefanova M. (2019). Could Dissimilar Phenotypic Effects of *ACTB* Missense Mutations Reflect the Actin Conformational Change? Two Novel Mutations and Literature Review. Mol. Syndromol..

[44] Nie M., Liu Q., Yan C. (2022). Skeletal Muscle Transcriptomic Comparison Between Men and Women in Response to Acute Sprint Exercise. Front. Genet..

[45] Weitensteiner V., Zhang R., Bungenberg J., Marks M., Gehlen J., Ralser D.J., Hilger A.C., Sharma A., Schumacher J., Gembruch U. (2018). Exome sequencing in syndromic brain malformations identifies novel mutations in ACTB, and SLC9A6, and suggests BAZ1A as a new candidate gene. Birth Defects Res..

[46] Rall N., Leon A., Gomez R., Daroca J., Lacassie Y. (2018). New ocular finding in Baraitser-Winter syndrome (BWS). Eur. J. Med. Genet..

[47] Nunoi H., Yamazaki T., Tsuchiya H., Kato S., Malech H.L., Matsuda I., Kanegasaki S. (1999). A heterozygous mutation of beta-actin associated with neutrophil dysfunction and recurrent infection. Proc. Natl. Acad. Sci. USA.

[48] Cuvertino S., Stuart H.M., Chandler K.E., Roberts N.A., Armstrong R., Bernardini L., Bhaskar S., Callewaert B., Clayton-Smith J., Davalillo C.H. (2017). ACTB Loss-of-Function Mutations Result in a Pleiotropic Developmental Disorder. Am. J. Hum. Genet..

[49] Lee C.G., Jang J., Jin H.S. (2018). A novel missense mutation in the ACTG1 gene in a family with congenital autosomal dominant deafness: A case report. Mol. Med. Rep..

[50] Wang H., Guan J., Lan L., Yu L., Xie L., Liu X., Yang J., Zhao C., Wang D., Wang Q. (2018). A novel de novo mutation of ACTG1 in two sporadic non-syndromic hearing loss cases. Sci. China Life Sci..

[51] De Heer A.M., Huygen P.L., Collin R.W., Oostrik J., Kremer H., Cremers C.W. (2009). Audiometric and vestibular features in a second Dutch DFNA20/26 family with a novel mutation in ACTG1. Ann. Otol. Rhinol. Laryngol..

[52] Liu P., Li H., Ren X., Mao H., Zhu Q., Zhu Z., Yang R., Yuan W., Liu J., Wang Q. (2008). Novel ACTG1 mutation causing autosomal dominant non-syndromic hearing impairment in a Chinese family. J. Genet. Genom..

[53] Yuan Y., Gao X., Huang B., Lu J., Wang G., Lin X., Qu Y., Dai P. (2016). Phenotypic Heterogeneity in a DFNA20/26 family segregating a novel ACTG1 mutation. BMC Genet..

